# Evaluating a Novel Simulation Course for Prehospital Provider Resuscitation Training in Botswana

**DOI:** 10.5811/westjem.2019.6.41639

**Published:** 2019-08-06

**Authors:** Adeola A. Kosoko, Nicolaus W. Glomb, Bushe Laba, Cafen Galapi, Manish I. Shah, Marideth C. Rus, Cara B. Doughty

**Affiliations:** * McGovern Medical School at the University of Texas Health Science Center at Houston (UTHealth), Houston, Texas; † Ministry of Health & Wellness, Emergency Medical Services System, Gaborone, Botswana; ‡ University of California, San Francisco School of Medicine, Department of Emergency Medicine, San Francisco, California; § Baylor College of Medicine/Texas Children’s Hospital, Department of Pediatrics, Houston, Texas

## Abstract

**Introduction:**

In 2012, Botswana embarked on an organized public approach to prehospital medicine. One goal of the Ministry of Health (MOH) was to improve provider education regarding patient stabilization and resuscitation. Simulation-based instruction is an effective educational strategy particularly for high-risk, low-frequency events. In collaboration with partners in the United States, the team created a short, simulation-based course to teach and update prehospital providers on common field responses in this resource-limited setting. The objective of this study was to evaluate an educational program for Botswanan prehospital providers via written and simulation-based examinations.

**Methods:**

We developed a two-day course based on a formal needs assessment and MOH leadership input. The subject matter of the simulation scenarios represented common calls to the prehospital system in Botswana. Didactic lectures and facilitated skills training were conducted by U.S. practitioners who also served as instructors for a rapid-cycle, deliberate practice simulation education model and simulation-based testing scenarios. Three courses, held in three cities in Botswana, were offered to off-duty MOH prehospital providers, and the participants were evaluated using written multiple-choice tests, videotaped traditional simulation scenarios, and self-efficacy surveys.

**Results:**

Collectively, 31 prehospital providers participated in the three courses. The mean scores on the written pretest were 67% (standard deviation [SD], 10) and 85% (SD, 7) on the post-test (p < 0.001). The mean scores for the simulation were 42% (SD, 14.2) on the pretest and 75% (SD, 11.3) on the post-test (p < 0.001). Moreover, the intraclass correlation coefficient scores between reviewers were highly correlated at 0.64 for single measures and 0.78 for average measures (p < 0.001 for both). Twenty-one participants (68%) considered the course “extremely useful.”

**Conclusion:**

Botswanan prehospital providers who participated in this course significantly improved in both written and simulation-based performance testing. General feedback from the participants indicated that the simulation scenarios were the most useful and enjoyable aspects of the course. These results suggest that this curriculum can be a useful educational tool for teaching and reinforcing prehospital care concepts in Botswana and may be adapted for use in other resource-limited settings.

## INTRODUCTION

Organized prehospital services in lower- and middle-income countries (LMIC) in sub-Saharan Africa continue to be in earlier stages of development compared to other regions worldwide. Only a minority of Africans (<9%) are covered by an emergency medical services (EMS) system.^1^ Implementation and development of an EMS system has had varied outcomes among LMICs. In 2012 the Ministry of Health (MOH) of Botswana established the country’s first public, prehospital EMS program.

At its inception, the Botswana public EMS program recruited most staff from MOH healthcare providers who were previously employed as nurses and healthcare attendants even though they did not have prior experience in prehospital care. A physician is the medical director of the public EMS system; however, physicians are not involved in the day-to-day work of the system. At the time of this study, the sole paramedic (trained internationally) in the EMS system served as its head of operations. Specific training, accreditation, and licensure requirements are necessary for a person to be identified as an emergency medical technician (EMT), either “basic” or “advanced.”

Boitekanelo College, a college that focuses on healthcare education in Gabarone, first started offering certificate diplomas and degree programs in EMS in 2011. However, few employees of the public EMS system were graduates of these programs at the time of this study, and there was no mandatory prehospital training for those newly employed by the system. At the time of this study, 115 EMS staff in Botswana (nurses, EMTs, healthcare assistants, and drivers) were stationed at six different EMS centers. As with many existing programs in sub-Saharan Africa, the Botswana EMS system offers primarily (but not always) basic life services, is financed and operated by the government, and has a public access telephone number for first response.

Poor outcomes in developing EMS systems are often due to a lack of resources, insufficient training, and other system deficiencies.^2^ The Botswana MOH has focused on optimizing the education and training of workers for initial patient stabilization and resuscitation. We created a curriculum designed to augment the training of prehospital care providers and enhance provider performance and patient outcomes. A critical step toward advancing prehospital care training in Botswana was to identify, establish, and promote sustainable instruments that were specifically suited to serve the local emergency medical conditions.^3^ Hence, we used the results of a formal needs assessment^4^ to better tailor an educational initiative.

The needs assessment of the Botswana MOH and Gaborone EMS system helped us to identify knowledge gaps and opportunities for educational development. Administrators and providers felt that prehospital providers were not optimizing opportunities for resuscitative interventions either in the field or en route to the hospital, partly because they were not familiar with supplies, lacked confidence in intervening, and failed to identify opportunities for intervention. The leading causes of EMS transport in the survey corresponded with the leading causes of EMS transport in Africa, namely, injury, obstetric, respiratory, cardiovascular, and gastrointestinal complaints.^1^ We found that medical simulation could be useful in addressing the needs of prehospital providers.

**Population Health Research Capsule**

What do we already know about this issue?

*Prehospital medical systems in low- and middle-income (LMIC) countries are actively being developed. Medical simulation has been shown to be an effective teaching tool*.

What was the research question?

*We examined whether a novel, simulation-based course would be an effective teaching tool for prehospital providers in Botswana*.

What was the major finding of the study?

*Over half of public Botswanan prehospital providers enrolled. Their test scores improved, and the course was well received*.

How does this improve population health?

*We hope to teach this course regularly in Botswana and believe it can be adapted for use in other LMICs to help improve the effectiveness of prehospital care*.

Simulation-based medical education enables providers to reproducibly practice high-risk scenarios in a safe learning environment. Simulation helps advance clinical knowledge, procedural skills, confidence, teamwork, and effective communication practices. The efficacy of this training tool for prehospital medicine has been established previously.^5^ Specifically, the rapid-cycle deliberate practice (RCDP) format was chosen for this particular population because it is well suited to those with less exposure to learning via the use of medical simulation and for those with the goal of attaining mastery.^6^ RCDP is an instructional method of simulation-based learning that combines multiple, shorter repetitions of cases with intermixed feedback and has been shown to improve key performance measures in resuscitation,^6^,^7^ specifically in teaching concepts of resuscitation in cardiac arrest, including assisted respiration, compressions, and defibrillation.^8^,^9^

We developed, implemented, and evaluated a simulation-based resuscitation curriculum for prehospital providers in Botswana. Outcomes included provider satisfaction with the curriculum and improvement of knowledge based on pre- and post-testing.

## METHODS

We developed a two-day, simulation-based training curriculum based on a formal needs assessment along with input from the Botswana MOH leadership. Simulation scenarios were based on the most frequent calls to the prehospital system, including abdominal pain, trauma, obstetric/gynecologic complications, respiratory distress, and weakness. The medical faculty from the U.S. presented supplementary didactic talks on how to approach medical simulation and a brief overview of approaching prehospital trauma specifically by request of the EMS administration. In addition, they conducted procedural-skills training sessions on intravenous/intraosseous access and oxygen delivery and instructed on RCDP simulation-based testing scenarios. The course was held in each of the three largest Botswanan cities: Gaborone, Francistown, and Mahalapye; it was offered to off-duty prehospital providers employed by the MOH. The participants were evaluated with written, multiple-choice tests, videotaped traditional simulation scenarios, and self-efficacy surveys administered before and after the training.

This study received institutional review board permission from the associated institutions both in the U.S. and the Botswana MOH.

### Study Population and Eligibility Criteria

Prehospital EMTs and nurses who were not on active duty during the training period were eligible to participate in the course (Table 1). The head of EMS requested that healthcare attendants and drivers should not participate in this training. In total, 31 (67.4%) of 46 prehospital providers in Botswana met the eligibility criteria and were included in the study. Due to the limited number of prehospital providers in the country, we decided not to have a comparison group for this study. However, to achieve the largest possible enrollment of off-duty providers, we offered the course three times in the three largest cities in Botswana.

### Data Management

We selected pairwise deletion as the most appropriate approach to address missing data. Specifically, for each analysis we included all observations with non-missing values for all variables relevant to that analysis. To enable quantitative analysis of self-efficacy survey data, Likert items were scored ranging from 1 for “extremely uncomfortable” to 7 for “extremely comfortable.”

### Statistical Analysis

#### Descriptive Statistics

Frequencies and percentages (for categorical variables) or medians and interquartile ranges (IQR) (for continuous variables) associated with demographic characteristics were calculated and reported.

#### Pre- vs Post-training Comparison

We compared continuous variables (written and simulation test scores) between two dependent groups (pre- and post-training) using the paired *t*-test, while ordinal variables (participant-reported, self-efficacy scores) were compared between two dependent groups (pre- and post-training) using the Wilcoxon signed-rank test.

#### Interclass Correlation

We used the Pearson correlation coefficient to determine interclass correlation between reviewers’ assessments of the simulation-based tests and associations between all three testing modalities (self-efficacy survey and written and simulation-based tests).

#### Assumptions and Tools

Hypothesis testing was considered statistically significant at *p* < 0.05. We performed all statistical analyses in Stata Statistical Software 15.1 (StataCorp 2017, College Station, TX, USA). Tables were computed using Microsoft Excel 2016 (Microsoft, Redmond, WA, USA).

## RESULTS

### Demographic Characteristics

Table 2 describes the participants’ demographic characteristics. Overall, 31 prehospital providers (19 [61%] male, 12 [39%] female) met the eligibility criteria and were included in the study. The participants were distributed roughly equally among the three study sites, including Francistown (10/31, 32.3%), Mahalapye (10/31, 32.3%), and Gaborone (11/31, 35.4%). The median number of years working in healthcare and in EMS was 6.0 years (IQR = 3.0–8.0) and 2.0 years (IQR = 1.0–2.0), respectively. The median number of self-reported, adult resuscitations performed in the past year was 1.0 (0–10 resuscitations). Prior to working in EMS, the participants had received training in Basic Life Support (20/31, 65%), Intermediate Life Support (10/31, 32%), Advanced Cardiovascular Life Support (3/31, 10%), and either Advanced Trauma Life Support or International Trauma Life Support (10/31, 32%). All certifications were reported based on published international and U.S. standards, as suggested.

### What is the impact of the curriculum on the self-reported self-efficacy of prehospital providers?

To determine the curriculum’s impact on the participants’ confidence in evaluating and managing adults with emergency conditions, we required that they complete a 14-item, self-efficacy survey before and after the training was implemented (Appendix A). The survey items were rated on a Likert scale from 1 (extremely uncomfortable) to 7 (extremely comfortable). Baseline self-efficacy scores are summarized in Table 3, and Table 4 compares the post-test scores.

### What is the impact of the curriculum on prehospital providers’ performance as measured by the written and simulation-based tests?

**Figure 1:**
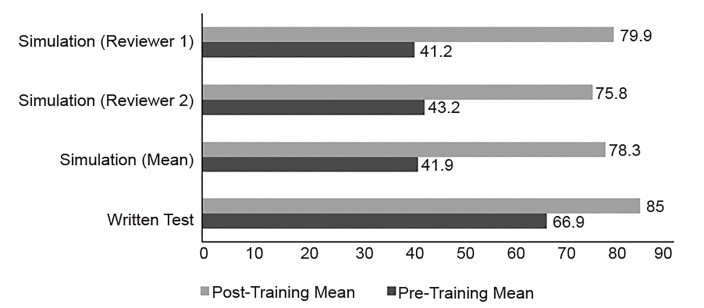
Participants’ written and simulation-based test scores before vs after training.

To evaluate the impact of the training curriculum on the participants’ knowledge and performance in evaluating and managing adults with emergency conditions, we required that they complete both the written (Appendix B) and simulation-based (Appendix C) tests before and after the training was implemented. The participants’ performance on each test was reported as a percentage. Table 5 and Figure 1 show the participants’ mean scores on both tests, before and after the training. Two reviewers independently rated each participant on the simulation test. The Pearson correlation coefficient was calculated to measure the interclass correlation between the two reviewers (Table 6).

### What is the association between participants’ written test scores, participant-reported self-efficacy, and performance on the simulation-based test?

To validate the written test score, we compared the participants’ scores on the written test to their self-efficacy scores and their simulation-based test scores using the Pearson correlation coefficient (Table 7). The pre-training written test scores and the pre-training simulation test scores had a moderate positive correlation (*r* = 0.41, *p* = 0.04). No significant correlation was observed between the corresponding post-training scores. Although the pre-training written score was positively correlated with the pre-training self-efficacy score, this finding was not statistically significant (*r* = 0.34, *p* = 0.06). In addition, we observed no significant correlation between the post-training written test and self-efficacy scores.

### Participant Feedback

The feedback was overwhelmingly positive with 100% of the participants reporting that the course was “useful.” In total, 21 participants (68%) answered that the course was “extremely useful” and the remaining 32% found the course “very useful.” The participants indicated that the best part of the course was the medical simulation, particularly the RCDP.

“Simulation as they gave real-life scenarios that we see every day.”

“Simulation, scenario, and giving feedback on how [we] performed on scenarios.”

“[My favorite part was] guided simulation when we would stop and do a post-mortem of the scenario.”

“The simulation part where you have the chance to stop and assess the case.”

The participants’ recommendations for improving the curriculum varied, but many requested a longer curriculum incorporating other teaching methods.

“More theory before we get to simulations.”

“They should add videos to their simulation but everything else was perfect.”

“The course should be longer (offered over a number of days) because there is a lot of material to cover.”

“Not enough time and next time should be more days to learn more things.”

Overall, the participants enjoyed the curriculum and reported that they would be able to incorporate what they had learned from the training into their clinical practice.

“Thank you for your time and teachings. I think I’m well equipped to manage the patient better than before.”

“I did have a great and fun time of learning, and I have certainly learned a lot from this course. [I] am going to use what I learned here to save lives.”

“… Course was informative and relevant.”

“… I have learned a lot from this training. Wish we could regularly do this kind of training.”

## DISCUSSION

Only limited information is available regarding the development of EMS systems of LMICs. However, based on published literature on LMIC EMS systems, an emphasis is often on transport, rather than on prehospital medical care.^10^ A disproportionate number of deaths occur outside the hospital in most LMICs compared to that in high-income countries.^11^ As in-hospital emergency care needs are being addressed internationally,^12^ efforts to increase the capacity and effectiveness of prehospital providers in LMICs, particularly in medical intervention, are warranted. We successfully developed and implemented a novel, simulation-based curriculum for prehospital providers in Botswana.^13^ To our knowledge, there are no similar training programs regularly used in LMICs to develop the skills of EMS providers.

A majority of providers had not undergone formal training to address prehospital patient care and medical intervention. Interestingly, the mean number of adult resuscitations in the past year (Table 2) of the study was one. Because this is self-reported information, it is unclear whether the providers were not correctly identifying interventions as resuscitations, whether they were adhering more to a “scoop and run” system (simply transporting the patients as fast as they could without emphasizing intervention), whether they abstained from resuscitative efforts due to lack of training, or whether there was some other reason for this low reported value. Nonetheless, participants’ prior experiences with resuscitation were low.

We trained 31/46 (67%) of the study-eligible providers in Botswana. Evaluations of the curriculum show that it was an appropriate, effective, and refreshing method of teaching prehospital providers resuscitation and stabilization skills. Overall, the participants reported improved self-efficacy in the topics covered and objectively demonstrated a statistically significant improvement in both written and simulation practical testing. Interestingly, the written test scores did not correspond significantly with self-reported efficacy or simulation test scores post-training (Table 7). Based on our results, a key future investigation would identify and investigate particular clinical outcomes to evaluate the participants’ theoretical knowledge (measured by written and simulation testing) measured against in-field performance.

The organizational structure of the Botswana EMS system is similar to many others in sub-Saharan Africa, and the field calls on which the simulation cases were developed correspond with the leading causes of EMS transport in Africa. Although this educational curriculum was specifically developed for use in Botswana, we believe that with minor adjustments it could be customized and applied in prehospital training in other LMIC countries in Africa and perhaps beyond.

This course is an educational tool that we plan to offer regularly throughout the major cities in Botswana as a refresher course for prehospital providers. In addition, our goal is to identify local Botswanan practitioners who have completed the course and are interested in teaching the curriculum independently without the curriculum authors or simulation specialists being present.

## LIMITATIONS

This study has a few limitations. First, the study group was a convenience sample based on the availability of EMS workers. As the pool of EMS providers in Botswana is relatively small, this led to a small sample size making it less prudent to compare intervention and control groups. In addition, many participants were dissatisfied with the short duration of the course and requested that it be longer and cover more topics. This is an opportunity to integrate and offer a concomitant simulation-based curriculum with this course. The course lasted for two days and evaluated pre- and post-testing one day apart. Retention of knowledge could have been re-evaluated several weeks or months after the course to obtain longer-term outcome data. We did our best to account for inter-rater variability when reviewing the video footage and subject evaluations; however, there is always potential for human error.

This was an educational study, which addressed the need for training; however, it did not address other system deficiencies. Similarly, Botswana is a middle-income country with limited resources designated for the EMS system. Although we considered the inventory of typical resources to design our education program, we did not evaluate how lack of resources affects the care provided. Rather, we focused on ensuring that the providers knew what resources were available to them and how they could be used. The focus of this study was not to evaluate the retention of knowledge, practice changes, or clinical outcomes resulting from this curriculum. However, researchers are currently evaluating whether the skills taught in this curriculum are affecting prehospital providers’ practice by reviewing patient report forms that note exactly what was done by prehospital providers in each actual patient field response. Preliminary results suggest that there has been a significant increase in the completion of tasks (evaluations and/or interventions).^14^ The researchers can also compare the actions of those who participated in the course with those who did not, and for those who did participate in this training, patient care interventions can be compared before and after the training.

## CONCLUSION

Prehospital medicine continues to develop and expand around the world, particularly in LMIC countries in sub-Saharan Africa. This simulation-based course was a novel and effective way to educate providers in Botswana on prehospital resuscitation. Future efforts should be directed toward evaluating longer-term retention of participant knowledge and evaluating behavioral changes of providers based on application of the curriculum concepts and how these applications affect patient outcomes. Although this study did not have a control group, future investigations could compare the patient outcomes of our course participants against those who did not participate considering that the participants were derived strictly from a pool of off-duty nurses and EMTs. At the time of this study, there was no formal training for Botswana EMS recruits. We plan to offer this course regularly; however, it is not compulsory. This curriculum could potentially be regularly used as an introductory course in prehospital resuscitation and as a refresher for those who may not be performing many prehospital, medical resuscitations in their practice.

The curriculum described in the present study represents a valuable educational tool that serves to educate healthcare providers, disseminate practical knowledge, and standardize clinical procedures. Implementing the concepts taught in this course could potentially advance prehospital medical care and patient outcomes not only in Botswana but also in other resource-limited environments.

## Supplementary Information







## Figures and Tables

**Table 1:** Total staffing within Botswana’s public emergency medical services system.

City	Emergency medical technicians	Registered nurses	Total
Francistown*	4	9	13
Gaborone*	5	8	13
Selebi-Phikwe	0	6	6
Mahalapye*	3	2	5
Palapye	0	5	5
Lobaste	0	4	4
Total	12	34	46

* Location of training.

**Table 2:** Characteristics of study participants in a course designed to improve prehospital care.

Characteristics	Frequency (%) N = 31
Sex	
Male	19 (61%)
Female	12 (39%)
Study site	
Francistown	10 (32%)
Gaborone	11 (35%)
Mahalapye	10 (32%)
Years in health care, median (IQR)	6.0 (3.0, 8.0)
Years in EMS, median (IQR)	2.0 (1.0, 2.0)
Adult resuscitations in the past year, median (IQR)	1.0 (1.0, 2.0)
Basic life support training	
No	11 (35%)
Yes	20 (65%)
Intermediate life support training	
No	21 (68%)
Yes	10 (32%)
Advanced cardiovascular life support training	
No	28 (90%)
Yes	3 (10%)
Advanced or international trauma life support training	
No	21 (68%)
Yes	10 (32%)

*IQR*, interquartile range; *EMS*, emergency medical services.

**Table 3:** Participants’ reported self-efficacy for various emergency medical services activities post-training.

Item	Rank	Pre-test frequency	Post-test frequency
Administering oxygen	Slightly comfortable	2 (6%)	
	Very comfortable	12 (39%)	6 (19%)
	Extremely comfortable	17 (55%)	25 (81%)
Placing an airway adjunct	Extremely uncomfortable	2 (6%)	
	Very uncomfortable	2 (6%)	1 (3%)
	Slightly uncomfortable	2 (6%)	
	Neutral	3 (10%)	
	Slightly comfortable	7 (23%)	1 (3%)
	Very comfortable	9 (29%)	7 (23%)
	Extremely comfortable	6 (19%)	25 (81%)
Administering rescue breaths with a BVM	Slightly comfortable	2 (6%)	
	Very comfortable	12 (39%)	6 (19%)
	Extremely comfortable	17 (55%)	25 (81%)
Managing an upper airway obstruction	Very uncomfortable	1 (3%)	
	Neutral	2 (7%)	1 (3%)
	Slightly comfortable	12 (40%)	2 (7%)
	Very comfortable	11 (37%)	8 (27%)
	Extremely comfortable	4 (13%)	19 (63%)
Recognizing signs of shock	Slightly comfortable	9 (29%)	
	Very comfortable	13 (42%)	5 (16%)
	Extremely comfortable	9 (29%)	26 (84%)
Providing fluid resuscitation	Neutral	1 (3%)	
	Slightly comfortable	4 (13%)	1 (3%)
	Very comfortable	16 (52%)	4 (13%)
	Extremely comfortable	10 (32%)	26 (84%)
Managing an adult with CHF	Extremely uncomfortable	1 (3%)	
	Very uncomfortable	1 (3%)	
	Slightly uncomfortable	3 (10%)	
	Neutral	8 (26%)	5 (17%)
	Slightly comfortable	13 (42%)	2 (7%)
	Very comfortable	5 (16%)	14 (47%)
	Extremely comfortable		9 (30%)
Ability to rapidly conduct a primary survey	Slightly comfortable	8 (27%)	1 (3%)
	Very comfortable	19 (63%)	10 (32%)
	Extremely comfortable	3 (10%)	20 (65%)
Immobilizing the cervical spine in trauma	Slightly uncomfortable	1 (3%)	
	Slightly comfortable	7 (23%)	1 (3%)
	Very comfortable	11 (35%)	8 (26%)
	Extremely comfortable	12 (39%)	22 (71%)
Managing a woman with vaginal bleeding	Slightly uncomfortable	1 (3%)	
	Neutral	2 (7%)	
	Slightly comfortable	3 (10%)	2 (7%)
	Very comfortable	18 (60%)	13 (43%)
	Extremely comfortable	6 (20%)	15 (50%)

*BVM*, bag-valve-mask; *CHF*, congestive heart failure.

**Table 4:** Participants’ reported self-efficacy scores before vs after training.

Variable	Pre-test median	Post-test median	P-value
Administering oxygen	7	7	0.01
Placing an airway adjunct	5	7	<0.001
Administering rescue breaths with a BVM	7	7	0.01
Managing an upper airway obstruction	5.5	7	<0.001
Recognizing signs of shock	6	7	<0.001
Providing fluid resuscitation	6	7	<0.001
Managing an adult with CHF	5	6	<0.001
Ability to rapidly conduct a primary survey	6	7	<0.001
Immobilizing the cervical spine in trauma	6	7	0.001
Managing a woman with vaginal bleeding	6	6.5	0.009

*BVM*, bag-valve-mask; *CHF*, congestive heart failure.

**Table 5:** Participants’ written and simulation-based test scores before vs after training.

Assessment	Pre-training mean % (SD)	Post-training mean % (SD)	Mean difference (SE)	P-value
Written test	66.9 (10.0)	85.0 (7.1)	18.0 (1.7)	<0.001
Simulation (reviewer 1)	41.2 (14.9)	79.9 (11.1)	38.7 (3.6)	<0.001
Simulation (reviewer 2)	43.2 (14.3)	75.8 (13.5)	32.7 (4.1)	<0.001
Simulation (mean)	41.9 (14.2)	78.3 (11.3)	36.3 (3.7)	<0.001

*SD*, standard deviation; *SE*, standard error.

**Table 6:** Interclass correlation between Reviewer 1 and Reviewer 2.

	Reviewer 1 (pre)	Reviewer 2 (pre)	Reviewer 1 (post)	Reviewer 2 (post)
Reviewer 1 (pre)	1.00			
Reviewer 2 (pre)	0.85***	1.00		
Reviewer 1 (post)	0.03	−0.14	1.00	
Reviewer 2 (post)	0.10	−0.11	0.76***	1.00

*Pre*, pre-training; *Post*, post-training.

* p < 0.05;

** p < 0.01;

*** p < 0.001

**Table 7:** Interclass correlation between written test scores, simulation-based test scores, and self-efficacy scores.

	Self-efficacy score (pre)	Self-efficacy score (post)	Written score (pre)	Written score (post)	Simulation score (pre)	Simulation score (post)
1Self-efficacy score (pre)	1.00					
1Self-efficacy score (post)	0.67***	1.00				
2Written score (pre)	0.34	0.33	1.00			
2Written score (post)	−0.13	−0.01	0.40*	1.00		
3Simulation score (pre)	0.47*	0.40*	0.41*	−0.03	1.00	
3Simulation score (post)	0.25	0.24	−0.19	−0.05	−0.07	1.00

* p < 0.05,

** p < 0.01,

*** p < 0.001

1 Mean score across items on participant-reported self-efficacy survey;

2 mean score on written test;

3 mean score across Reviewers 1 and 2 on simulation-based scenarios.
