# Supplementary material for: Evaluating a Novel Simulation Course for Prehospital Provider Resuscitation Training in Botswana
Source: West J Emerg Med. 2019 Aug 6;20(5):731–9. doi: 10.5811/westjem.2019.6.41639 (PMC6754192; doi:10.5811/westjem.2019.6.41639)
Supplement: Supplementary file 1 [file wjem-20-731-s001.docx]

**Pretest Questionnaire for Botswana EMS Simulation Training**

**The following questions ask for your level of confidence in performing the following skills. Please choose the answer that best matches your current level of confidence.**

**Prehospital Pretest Checklist** NAME_____________________________

**On a scale of 1 to 7 (see below), please indicate your LEVEL of COMFORT prior to the training using your knowledge and/or skills in the following aspects of prehospital care:**

| **1** | **2** | **3** | **4** | **5** | **6** | **7** | **Not Applicable** |
| --- | --- | --- | --- | --- | --- | --- | --- |
| **Extremely Uncomfortable** | **Very Uncomfortable** | **Slightly Uncomfortable** | **Neutral** | **Slightly Comfortable** | **Very comfortable** | **Extremely Comfortable** | **Not in my scope of practice** |

1. Administering oxygen (Examples: nasal cannula, face mask)

1 2 3 4 5 6 7 NA

1. Inserting an airway adjunct (Examples: nasopharyngeal or oropharyngeal airway)

1 2 3 4 5 6 7 NA

1. Administering rescue breaths with a bag-valve-mask (BVM)

1 2 3 4 5 6 7 NA

1. Managing an upper airway obstruction

1 2 3 4 5 6 7 NA

7. Recognizing the signs of shock

1 2 3 4 5 6 7 NA

8. Providing fluid resuscitation to patients presenting with shock

1 2 3 4 5 6 7 NA

10. Managing an adult with congestive heart failure

1 2 3 4 5 6 7 NA

13. Ability to rapidly conduct a primary survey on a trauma patient

1 2 3 4 5 6 7 NA

14. Immobilizing the cervical spine for trauma evaluation

1 2 3 4 5 6 7 NA

16. Managing a woman with vaginal bleeding

1 2 3 4 5 6 7 NA
