# Supplementary material for: Evaluating a Novel Simulation Course for Prehospital Provider Resuscitation Training in Botswana
Source: West J Emerg Med. 2019 Aug 6;20(5):731–9. doi: 10.5811/westjem.2019.6.41639 (PMC6754192; doi:10.5811/westjem.2019.6.41639)
Supplement: Supplementary file 2 [file wjem-20-731-s002.docx]

**Written Pretest**

1. You have been called to the home of a 20-year-old female with complaints of vaginal bleeding for the past 24 hours. In which of the scenarios listed below is HIV most likely to be transmitted?

a. Getting scratched by the woman

b. Wiping the woman’s tears with your bare hand

c. Getting blood in an open wound

d. Getting bit by the woman

2. You pick up a patient from a local school who is on a non-rebreather mask for respiratory distress. What should you do to deliver the highest concentration of oxygen?

a. Nasal cannula with 2 L/min oxygen flow

b. Simple face mask with 15 L/min oxygen flow

c. Non-rebreather facemask with 12 L/min oxygen flow

d. Bag-valve-mask 1 cm from the face with 15 L/min oxygen flow

3. You are assessing an adult male who was involved in a motor vehicle collision. He only opens his eyes to painful stimuli, speaks unintelligibly, and withdraws from painful stimuli. What is the Glasgow Coma Score for this patient?

a. 7

b. 8

c. 9

d. 10

4. A 60-year-old man with a history of heart disease has called for help because he has been feeling worse each day and now feels like he can't walk due to weakness. Signs of circulatory overload include:

a. Jugular venous distention, foot/leg edema, and difficulty breathing

b. Slow heart rate and blue extremities

c. Fast heart rate and unequal pupils

d. Slow heart rate and hypovolemia

5. Which of the following is the best indicator of successful intraosseous needle placement?

a. Fluids can be administered easily without local soft tissue swelling

b. The hub of the needle is touching the skin

c. You are able to aspirate blood from the needle

d. Pulsatile blood flow is present in the needle hub

6. A college student with a history of asthma has collapsed while walking home from the library. All of the following are signs of adequate breathing EXCEPT:

a. Audible breath sounds on both sides of the chest

b. Nasal flaring

c. Warm, dry skin without pallor

d. Respiratory rate of 18 breaths/min

7. A patient has tachycardia and confusion. A condition in which low blood volume resulting in inadequate perfusion is referred to as:

a. Hyperbole

b. Hypoxia

c. Hypovolemic shock

d. Hemorrhage

8. A patient has had vomiting and diarrhea for the past 3 days. An IV is started and fluids administered. Fluid resuscitation should be given using a(n):

a. Hypertonic solution

b. Isotonic solution

c. Hypotonic solution

d. Glucose-containing solution

9. You have been called to the home of a 75-year-old woman. Her son explains that she has felt tired and generally unwell for the past several hours. He denies that she has any cardiac history. True or False: All patients experiencing a myocardial infarction will present with chest pain.

a. True

b. False

10. An obese man is found unconscious in his home. A friend discovered him after going to check on him and was not able to wake him. You perform an initial assessment. Often the most effective initial airway management technique is:

a. Placing an oropharyngeal airway

b. Placing an oxygen mask at 10 L/min flow rate

c. Suctioning the airway and providing assistance with a bag-valve-mask

d. Using a head-tilt/chin-lift or jaw thrust maneuver

11. A woman has presented with significant vaginal bleeding and pelvic pain lasting for the past several hours. You should:

a. Cover the vagina with a feminine pad

b. Pack the vagina with a trauma dressing

c. Apply direct pressure to control the bleeding

d. Elevate the patient’s pelvis

12. You are checking the vital signs of a 38-year-old woman who had a syncopal episode. Vital signs are most comprehensive when the following are measured:

a. Pulse rate (rhythm and quality), temperature, and pulse oximetry

b. Temperature, blood pressure, pulse oximetry, and respiratory rate

c. Pulse rate (rhythm and quality), respiratory rate (rhythm and quality), blood pressure,

temperature, and pulse oximetry

d. Pulse rate, blood pressure, breath sounds, and temperature

13. A 25-year-old farmer has called for help because he has had sudden increased difficulty breathing. What are you listening for when listening to his lungs?

a. Normal breath sounds, or wheezes, rales, rhonchi, or stridor

b. Normal lung sounds or fluid in the lungs, asthma, coughing

c. Normal lung sounds, emphysema, pneumonia, wheezing

d. Normal lung sounds, decreased breath sounds, decreased tidal volume

14. Diarrhea or hemorrhage can cause an absolute loss of fluid volume leading to:

a. Infection

b. Shock

c. Hypertension

d. Chills

15. A patient has jumped from a building. After 2 attempts at a peripheral IV for fluid resuscitation, you are considering placing an intraosseous needle. Intraosseous access is contraindicated in:

a. The elderly

b. Children

c. The awake patient

d. A bone with a known or suspected fracture
