# Supplementary material for: Evaluating a Novel Simulation Course for Prehospital Provider Resuscitation Training in Botswana
Source: West J Emerg Med. 2019 Aug 6;20(5):731–9. doi: 10.5811/westjem.2019.6.41639 (PMC6754192; doi:10.5811/westjem.2019.6.41639)
Supplement: Supplementary file 3 [file wjem-20-731-s003.docx]

**Trauma Team Evaluation Tool**

| City: | Date/Day | PRE or POST | Total time: minutes |
| --- | --- | --- | --- |
| Trainee: | | | |
| Evaluator: | | | |
| Scenario: Trauma | | | |

| **I. Team Organization** | |
| --- | --- |
| 1. Verbal communication within the team | 0 = no clear instructions |
|  | 1 = somewhat clear communication or inconsistent communication |
|  | 2 = consistently clear communication |
| 1. Systematic and orderly assessment | 0 = disorganized or incomplete primary survey |
|  |  |
|  | 2 = organized and complete primary survey |
| 1. Ability to handle distractions | 0 = allowed attention to be diverted and addressed before addressing A, B, and C |
|  |  |
|  | 2 = addressed the distractor at the appropriate time |

| **II. Airway and Breathing** | |
| --- | --- |
| 1. Airway assessed | 0 = no assessment |
|  |  |
|  | 2 = airway assessed |
| 1. Oxygen administered | 0 = no oxygen delivered |
|  | 1 = delivered oxygen, but not during airway or breathing evaluation |
|  | 2 = delivered oxygen during airway or breathing evaluation |
| 1. Time to assess airway and breathing (from initial interaction with patient) | 0 = >60 seconds |
|  | 1 = 30–60 seconds |
|  | 2 = <30 seconds |

| **III. Circulation** | |
| --- | --- |
| 1. Initiated BP check | 0 = >3 minutes |
|  | 1 = 2–3 minutes |
|  | 2 = <2 minutes |
| 1. Initiated HR check | 0 = >3 minutes |
|  | 1 = 2–3 minutes |
|  | 2 = <2 minutes |
| 1. Assessed for central or peripheral pulses during primary survey | 0 = not checked |
|  |  |
|  | 2 = checked |
| 1. Time to achieve IV access | 0 = >5 minutes or none established |
|  | 1 = 3–5minutes |
|  | 2 = <3 minutes |
| 1. Administered IV fluids for possible shock | 0 = no fluids given |
|  | 1 = low volume or slow rate of NS or LR (“slow,” “drops”) |
|  | 2 = 1 liter of NS or LR at rapid rate (“push/pull,” “fast,” “wide open,” or “<15 min”) |
| 1. Timely initiation of IV fluids | 0 = >6 minutes |
|  | 1 = 4–6 minutes |
|  | 2 = <4 minutes |

| **IV. Disability** | |
| --- | --- |
| 1. Assessed responsiveness | 0 = no |
|  |  |
|  | 2 = yes |
| 1. Assessed pupils | 0 = not examined |
|  |  |
|  | 2 = examined |
| 1. Took C-spine precautions | 0 = none |
|  | 1 = performed improperly or in an untimely manner |
|  | 2 = airway assessment performed properly |
| 1. Objective measurement of mental status | 0 = none performed |
|  | 1 = incorrect assessment of GCS or AVPU |
|  | 2 = correct measurement of GCS or AVPU |
| 1. Splinted a broken extremity | 0 = no immobilization |
|  |  |
|  | 2 = immobilized affected extremity |

| **V. Exposure** | |
| --- | --- |
| 1. Performed a log roll | 0 = not performed |
|  |  |
|  | 2 = performed properly with C-spine immobilization |
| 1. Exposed patient for further evaluation | 0 = not performed |
|  |  |
|  | 2 = attempted to look at covered areas |

| **VI. Total Score** | |
| --- | --- |
| 1. Total Points |  |
| 1. Total Points Possible | 38 |
| 1. Percentage | % |
